# Pathogenesis of human-derived *Bacillus cereus* strains: lessons from the insect *Galleria mellonella* immune responses

**DOI:** 10.3389/fcimb.2026.1698447

**Published:** 2026-04-15

**Authors:** Jessica Minnaard, Fernando Miguel Trejo, Christophe Buisson, Alexander Bolotin, Vincent Sanchis-Borja, Christina Nielsen-LeRoux, Pablo Fernando Pérez

**Affiliations:** 1 Centro de Investigación y Desarrollo en Ciencia y Tecnología de los Alimentos, Centro Científico Tecnológico-CONICET, La Plata, Argentina; 2 Cátedra de Microbiología, Facultad de Ciencias Exactas, Universidad Nacional de La Plata, La Plata, Argentina; 3 Instituto de Ciencias de la Salud (Universidad Nacional Arturo Jauretche-ICS), Universidad Nacional Arturo Jauretche, Florencio Varela, Argentina; 4 Université Paris Saclay, Institut National de Recherche pour l’Agriculture, l’Alimentation et l’Environnement, AgroParisTech, Micalis Institute, Jouy-en-Josas, France

**Keywords:** *Bacillus cereus*, food-borne pathogens, *Galleria mellonella*, insect model, pathogenesis

## Abstract

**Introduction:**

*Bacillus cereus* is responsible for a wide range of intestinal and extraintestinal infections in humans. Its pathogenicity relies on multiple factors, including extracellular toxins, direct interaction with host tissues, and adaptive mechanisms that promote host colonization. *B. cereus* group bacteria are also insect pathogens (e.g., *Bacillus thuringiensis*), suggesting that certain virulence mechanisms may be conserved between mammals and insects.

**Methods:**

In this study, we used *Galleria mellonella* as an infection model to assess the pathogenicity of two *B. cereus* strains (i.e., T1 and B10502), which were previously isolated from food poisoning outbreaks and that differ in their virulence toward human enterocyte cell cultures. We combined genomic analysis with larval infection assays to examine survival, bacterial persistence, immune activation, and spore formation.

**Results:**

Whole-genome phylogenetic analysis revealed that the two strains belong to distinct branches of the *B. cereus sensu lato* species. Both strains induced dose-dependent mortality following oral gavage, with strain T1 showing a better persistence than strain B10502 in both living and dead larvae, with heat-resistant spores detectable up to 144 h post-infection, unlike strain B10502. Infection with strain B10502 elicited higher phenoloxidase activity and greater melanization than with strain T1. Both strains similarly reduced hemocyte viability.

**Discussion/conclusion:**

Genomic comparisons revealed that both strains share a core set of virulence factors, including the non-hemolytic enterotoxin (Nhe) complex, various hemolysins, and phospholipases, while exhibiting significant differences in genes, such as *hblABCD* complex, *mpbE*, *clpC*, *clpP*, and *ilsA*. These findings demonstrate that *G. mellonella* is a useful infection model to discriminate *B. cereus* strains with different virulence biological activities on larval colonization and innate immune markers, providing new insights into the mechanisms underlying the pathogenicity of foodborne *B. cereus* strains.

## Introduction

*Bacillus cereus* is a spore-forming opportunistic pathogen associated with both intestinal and non-intestinal human pathologies (Bottone, 2010; Enosi Tuipulotu et al., 2021).

In humans, *B. cereus* is responsible for different pathologies, including emetic and diarrheic syndromes (Stenfors Arnesen et al., 2008; Bottone, 2010; Jovanovic et al., 2021), endophthalmitis (Moyer et al., 2008; Mursalin et al., 2020), endocarditis (Castedo et al., 1999; Thomas et al., 2012; Ehrenzeller et al., 2025), osteomyelitis, nosocomial bloodstream infections (Glasset et al., 2018), oral cavity infections (Kotiranta et al., 2000), septicemia (Rowan et al., 2001), peritonitis (Drewett et al., 2018), pneumonia (Wright et al., 2011; Miyata et al., 2013), and meningitis (Stevens et al., 2012). Although the majority of cases are mild, life-threatening infections have been reported (Akiyama et al., 1997; Mahler et al., 1997; Naranjo et al., 2011; Wright et al., 2011; Tschiedel et al., 2015; Qiu et al., 2019; Jessberger et al., 2020), highlighting the need to better understand the mechanisms governing the pathogenicity of *B. cereus*.

Following ingestion, *B. cereus* spores germinate in the digestive tract, and vegetative cells release different extracellular factors with biological activity, including hemolysins, phospholipases, and cytotoxins (Lund and Granum, 1997; Beecher and Wong, 2000; Stenfors Arnesen et al., 2008; Jovanovic et al., 2021). The major virulence factors include the tripartite toxin complexes hemolysin BL (Hbl) and non-hemolytic enterotoxin (Nhe), cytotoxin K (CytK), phospholipases, and sphingomyelinases. Hbl and Nhe cause pore formation in host cell membranes, leading to cytotoxicity, while CytK exhibits necrotic activity (Jovanovic et al., 2021). Phospholipase C contributes to tissue damage and immune evasion (Oda et al., 2012), and bacterial surface proteins such as IlsA facilitate iron acquisition in iron-limited host environments (Daou et al., 2009). In addition, *B. cereus* can adhere to and invade epithelial cells, suggesting that direct bacterial–host cell interactions can also contribute to its virulence (Minnaard et al., 2004). Genetic diversity among strains has also been linked to differences in toxin expression and pathogenic potential (Minnaard et al., 2001, 2004).

The remarkable ecological versatility of *B. cereus* group members, which can adopt saprophytic, symbiotic, or pathogenic lifestyles depending on the strain, environmental conditions, and host species, poses significant challenges for dissecting their virulence mechanisms (Ceuppens et al., 2012). While the majority of *in vivo* studies on the effect of bacteria belonging to the *B. cereus* group have focused on *Bacillus anthracis* (Heninger et al., 2006; Glomski et al., 2007; Xie et al., 2013; Gao et al., 2024; Jiranantasak et al., 2024), fewer studies have explored gastrointestinal infection models for *B. cereus.* A murine model of gastrointestinal infection by *B. cereus* has been established (Rolny et al., 2014) and gut colonization has been documented in rats (Wilcks et al., 2006). The use of alternative *in vivo* systems such as *Galleria mellonella*, the larval stage of the greater wax moth, provides a valuable bridge between *in vitro* assays and vertebrate models. *G. mellonella* possesses an innate immune system that shares significant functional parallels with the mammalian response, comprising both cellular and humoral components (Kavanagh and Reeves, 2004). This multifaceted nature allows for the assessment of host–pathogen interactions under physiologically relevant conditions. Specifically, the model facilitates the simultaneous study of humoral defenses [including melanization, phenoloxidase (PO) activity, and antimicrobial peptides] and the cellular responses mediated by hemocytes, providing deep insights into the conserved mechanisms of innate immunity (Upfold et al., 2023).

In the present study, *G. mellonella* was used to assess and compare the virulence potential of two *B. cereus* strains, i.e., B10502 and T1, which were previously characterized for their distinct biological activities in human cell culture models (Minnaard et al., 2001, 2004, 2013; Rolny et al., 2017). Strain B10502 was involved in a food poisoning outbreak in Argentina (Minnaard et al., 2001) that affected approximately 400 people, the majority of whom were elderly. Patients presented with classic gastrointestinal symptoms, and the strain was isolated from a mayonnaise sample. The isolate T1 was implicated in a food poisoning outbreak estimated to have occurred in the 1990s (Buchanan and Schultz, 1994). By combining infection assays with genomic and immunological analyses, we aimed to identify strain-specific host–pathogen interactions and to evaluate the usefulness of *G. mellonella* as a model for investigating the pathogenicity and immune evasion strategies of *B. cereus*.

## Materials and methods

### *Bacillus cereus* strains and culture conditions

*The B. cereus* strains T1 and B10502, which have been associated with foodborne outbreaks and previously characterized for their differential virulence toward human enterocytes, were used in this study (Minnaard et al., 2007, 2013). Stock cultures were prepared by growing bacteria in brain heart infusion (BHI) broth (BD BACTO™; Becton, Dickinson and Company, Franklin Lakes, NJ, USA) to mid-log phase, supplemented with 20% (*v*/*v*) sterile glycerol as cryoprotectant, and stored at −80°C until use. Strains were reactivated by streaking frozen stocks onto Luria–Bertani (LB) agar plates (BD Difco™; Becton, Dickinson and Company, Franklin Lakes, NJ, USA) and incubating at 37°C for 16 h. Single colonies were inoculated into LB broth (initial OD_600_ = 0.01) and cultured with shaking (180 rpm) at 37°C until the mid-exponential growth phase (OD_600_ = 1–2). Cultures were harvested by centrifugation (5,000 × *g*, 5 min) and the bacteria suspended in sterile saline solution (8.5 g/L NaCl), which were used for infection studies. To perform experiments, the bacterial concentrations were estimated by means of a calibration curve [colony forming units (CFU) per milliliter *vs*. OD_600_]. In parallel, the actual viable counts were determined following serial dilutions and plate counts on LB agar.

### Phylogeny and genomic analysis

Complete genome sequencing of B10502 and T1 was performed using Illumina technology (San Diego, CA, USA). For genomic sequencing, total DNA of strains B10502 and T1 was prepared using water-saturated phenol treatment. Standard genomic libraries and sequencing reads were produced by Eurofins GATC Biotech GmbH (Konstanz, Germany) using a HiSeq 3000/4000 platform (Illumina, San Diego, CA, USA). Template-independent genomic sequences were obtained using 9,236,834 and 9,318,205 paired-end 150-base-long reads (maximum read length = 126) for B10502 and T1, respectively. *De novo* assembly done with Unicycler version 0.4.8 (Wick et al., 2017) provided 51 (5,221,394 bp in total, mean coverage = 243) and 39 (5,188,445 bp in total, mean coverage = 254) contiguous sequences longer than 200 bases for T1 and B10502, respectively.

The MinHash MASH v.2.3 (Ondov et al., 2016) and skani (0.2.1) of GTDB-Tk toolkit (2.4.0) (Chaumeil et al., 2020) were used to compare the B10502 and T1 assemblies to the GenBank REFSEQ bacterial genomes and to define the phylogenetic and taxonomic emplacements inside the group.

The Prokka 1.14.6 (Seemann, 2014) and Bakta 1.9.1 (Schwengers et al., 2021) pipelines were used for genome sequence annotations. Whole-genome clustering was performed and phylogenetic relationships were determined using Average Nucleotide Identity (ANI) distance estimation software by pyani (0.2.11), skani (0.2.1), and BTyper3 version 3.4.1 (Carroll et al., 2020). The genomes of strains B10502 and T1, as well as sets of the best matching and representative genomes of the *B. cereus sensu lato* species in GTDB (Chaumeil et al., 2020) (dataset in **Supplementary Material**), were utilized in further comparative genome analysis and pangenome construction using PPanGGOLiN (2.0.4) (Gautreau et al., 2020).

Prediction of virulence-related gene candidates was performed with several approaches, including Abricate (version 1.0.1; Seemann T, *Abricate*, Github VFDB dataset) (Chen et al., 2016), BTyper3 (default parameters, internal VF dataset), and the PPanGGOLiN pangenome analysis suite with a combined set of VFDB+BTyper3 and an additional subset of selected virulence and adaptation genes, which were exploited to improve pipeline-generated genome annotation. The MIGALE (https://migale.inrae.fr) INRAE reference platform (LRQA certificate identity no. 10531223; ISO 9001-0033333) in the field of bioinformatics and provision of an infrastructure for calculation, storage, public data, and tools dedicated to the processing of life science data were exploited in this publication.

Data on BioProject PRJNA1115104 are accessible at https://www.ncbi.nlm.nih.gov/sra/PRJNA1115104. The raw Illumina sequence data for the *B. cereus* strains B10502 and T1 are available in the NCBI-SRA (Short Read Archive) repository under accession numbers SRR34420510 and SRR34420511, respectively. The Whole Genome Shotgun project has been deposited at DDBJ/ENA/GenBank with accession numbers JBPPSQ000000000 for *B. cereus* B10502 and JBPPSP000000000 for *B. cereus* T1. The versions described in this paper are versions JBPPSQ010000000 and JBPPSP010000000, respectively.

### *Galleria mellonella* infection protocol

Infection assays were performed on last-instar *G. mellonella* larvae (250 mg), reared in the laboratory (INRAE, Jouy-en-Josas, France) on pollen and bee wax (La Ruche Roannaise Besachier, Roanne, France). For infection, groups of 20 larvae (250–300 mg each) were used for each treatment. Larvae were inoculated with bacterial suspensions prepared as described above. Infections were performed by force feeding (gavage) with 10 µl of different doses of bacterial suspensions (between 5.5 and 8 log_10_ CFU/larva). The Cry1Ca activated toxin from *Bacillus thuringiensis* was co-administered at 3 µg/larva to facilitate gut epithelial disruption. Each infection was performed in triplicate. Force feeding was done using hypodermic needles, 30 G–25 mm (Burkard, Hertfordshire, England), coupled to an injector pump, KDS100 (KD Scientific, Thermo Fisher, Illkirch, France). Control larvae were administered with the Cry1Ca toxin (3 µg/larva) or phosphate-buffered saline (PBS). Afterward, the larvae were maintained at 37°C. The ratio of dead larvae and the melanization score of each larva were evaluated at different time intervals. Melanization scores were defined as 0 (non-melanized, similar to controls), 2 for black larvae fully melanized, and 1 for larvae between 0 and 2.

#### Assessment of bacterial loads in the larvae and spore enumeration

The colonization kinetics was analyzed at 24, 48, 96, and 144 h post-infection (PI). The analysis of bacterial persistence in larvae was performed on dead larvae at 24, 48, and 144 h PI. Dead larvae were maintained at 37°C until analysis. For each condition and time point, two larvae were analyzed. Larvae were surface-disinfected by immersion in 70% (*v*/*v*) ethanol for 20 s. Excess liquid was then removed using sterile absorbent paper. Larvae were then homogenized for 30 s (ULTRA-TURRAX T-50) IKA, Germany in 10 ml sterile PBS buffer, and serial dilutions were plated on LB agar for CFU counts. Subsequently, appropriate dilutions were plated onto BHI agar and incubated at 37°C. The presence of thermoresistant spores was quantified by heating homogenates at 65°C for 30 min before plating. To minimize interference from larval gut microbiota, CFU counts were performed after less than 24 h to count the *B. cereus* colonies only, before outgrowth of eventual larval gut microbiota (Upfold et al., 2023). It is worth noting that uninfected controls showed no *B. cereus*-like colonies. Furthermore, no heat-resistant colonies—other than our inoculated strain—were recovered from the larval homogenates after heat treatment.

#### Analysis of hemocoel

##### Hemolymph obtention

Before manipulation, the larvae were maintained on ice for 5 min. The surface of the larvae were disinfected by immersion in ethanol (70%, *v*/*v*). Thereafter, the cuticle was punctured with a needle for hemolymph recovery. To prevent coagulation and melanization, 20 µl of hemolymph was collected on 480 µl of ice-cold anticoagulant buffer (KCl, 69 mM; NaCl, 27 mM; NaHCO_3_, 2 mM; d-glucose, 100 mM; citrate triphosphate, 30 mM; citric acid, 26 mM; EDTA-Na_2_, 10 mM) as described previously (Admella and Torrents, 2022).

##### Melanization scoring and phenoloxidase activity in hemolymph

Melanization of larvae was scored 24 h PI using a 0–2 scale: 0 = no melanization; 1 = partial; 2 = full melanization (Loh et al., 2013). The PO activity in hemolymph was measured 4 h PI using l-DOPA substrate as described previously (Ryazanova et al., 2012). The hemolymph samples, obtained from live larvae, were centrifuged for 5 min at 7,000 × *g* and 4°C, and 20 µl of the hemolymph supernatants was mixed with 20 µl of l-DOPA (3,4-dihydroxyphenylalanin, 2 mg/ml) and 80 µl of PBS in a 96-well microplate. The absorbance (*λ*_490_) was recorded every 5 min for 90 min in a Tecan Infinite M PLEX plate reader (Thermo Fisher Scientific, Lyon, France). Phenol oxidase activity was calculated as the initial slope of the plot absorbance per milligram protein *vs*. time. The protein content was analyzed with the Bradford method (Bradford, 1976) using a colorimetric assay (Bio-Rad Laboratories, Inc., Hercules, CA, USA) according to the manufacturer’s instructions.

##### Hemocyte viability assay

Circulating hemocytes were quantified 4 h PI with trypan blue staining and counting to evaluate the number of viable hemocytes. Larvae were maintained on ice for 5 min. Hemolymph was collected after picking the cuticle with a 25-G needle, and 20 µl was placed into 480 µl of Grace’s insect medium (Sigma, St. Louis, MO, USA). The cells recovered after centrifugation (5 min, 700 × *g*) were suspended in 200 µl of Grace’s medium and analyzed using light microscopy in a counting chamber (KOVA Glasstic Slide 10, Gardan Grove, CA, USA). All assays were performed in triplicate.

### Statistical analysis

Statistical differences between means were assessed with a two-tailed Student’s *t*-test. Melanization of larvae was analyzed using the Bonferroni multiple test. Differences between proportions (i.e., mortality of larvae) were analyzed with Fischer’s exact test. Kinetics of the survival of larvae were assessed by means of the Kaplan–Meier estimate and analyzed using Gehan–Breslow–Wilcoxon.

Phylogenetic analysis was performed on the basis of the genomic sequences of the *B. cereus* group strains using the Mash toolkit (Ondov et al., 2016).

Log_10_ of effective dose 50 (Log ED_50_) was calculated by probit analysis using cumulative mortality values (in percent).

## Results

### Genomic analysis of the *B. cereus* strains B10502 and T1

**Figure 1:**
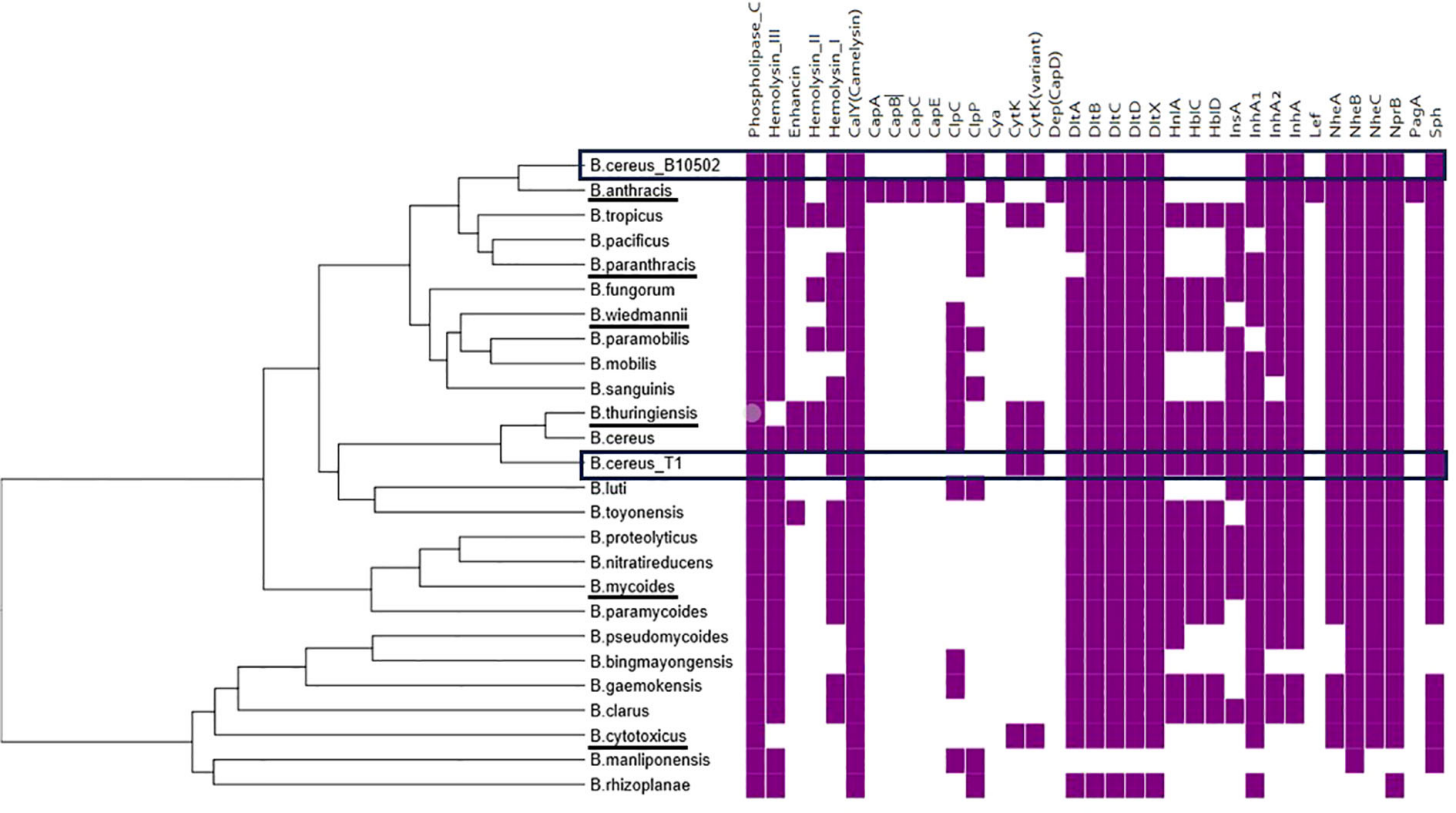
Phylogenetic position of the B10502 and T1 strains within the updated list of 24 *Bacillus cereus sensu lato* species and the presence/absence of a subset of selected virulence or adaptation genes relevant to pathogenesis in mammalian and insect models. A whole-genome clustering approach based on Average Nucleotide Identity (ANI) distance estimations was used. Underlined species have been reported in human infections.

The two *B. cereus* strains studied in this work, B10502 and T1, were previously analyzed *in vitro* using cultured eukaryotic cells. To understand the phylogenetic relationship and the genetic basis for potential virulence differences between strains B10502 and T1, we performed whole-genome sequencing (WGS) and comparative genomic analysis. Whole-genome assemblies of strains B10502 and T1 were compared to reference genomes from GenBank RefSeq to compute the genetic distances between the two sequences relative to the bacterial genomes in the GenBank RefSeq database. Genome annotations were performed using multiple bioinformatic pipelines. ANI-based distance estimations enabled clustering and phylogenetic comparison of the B10502 and T1 genomes with reference genomes of the *B. cereus sensu lato* group. **Figure 1** shows the phylogenetic position of the strains under study. In addition, further information on the functional groups of genes is provided in **Supplementary Table 1**.

Beyond the well-established pathogens *B. cereus* and *B. anthracis*, several other members of the *B. cereus sensu lato* group have been increasingly recognized as significant human pathogens. *B. thuringiensis*, traditionally known as a biopesticide, has been implicated in severe opportunistic infections, including fatal sepsis and endophthalmitis (Ghelardi et al., 2007; Butcher et al., 2021). Similarly, the thermotolerant species *Bacillus cytotoxicus* is associated with severe food poisoning due to its production of the potent CytK-1 toxin (Guinebretière et al., 2013), and *Bacillus mycoides* is responsible for bloodstream infection (Heidt et al., 2019). Recent advancements in WGS have further expanded this clinical spectrum, identifying emerging species such as *Bacillus paranthracis* in cases of bacteremia (Wang et al., 2024) and cytotoxic *Bacillus wiedmannii* strains (Miller et al., 2016). These findings emphasize the clinical relevance of the group’s broader taxonomic diversity and the necessity for precise identification beyond traditional species boundaries (Carroll et al., 2022).

Based on phylogenetic analysis, strain B10502 is closer to *B. anthracis*, whereasstrain T1 clusters more closely with *B. cereus/B. thuringiensis* (**Supplementary Figure 1**).

To explore the presence of virulence and the host adaptation factors relevant to insect pathogenesis, both genomes were screened for genes encoding hemolysins, enterotoxins (*hbl* and *nhe* complexes), phospholipase C (*plc*), sphingomyelinase (*sph*), Clip proteases (*clpP* and *clpC*), surface proteins involved in host iron acquisition (*ilsA*), resistance to antimicrobial peptides (*dltXABCD* operon), or metalloproteases inhibiting immune resistance (*inhA*’s) (**Figure 1**, **Supplementary Figure 2**). This survey both confirmed and extended our previous PCR-based screening (Minnaard et al., 2007), which identified the presence of specific enterotoxin and phospholipase genes. The WGS provided complete virulence gene profiles and revealed additional differences. This analysis indicated that both B10502 and T1 strains contain *plc*, the *dlt* operon, the *nhe* complex, and the *sph*, *calY* (Diehl et al., 2026), and *inhA* genes, as well as *cytK*, a gene encoding cytotoxin K that is relatively rare among the 24 type strains of the *B. cereus sensu lato* group. Other genes, e.g., *nprB* (Pomerantsev et al., 2011), a neutral protease involved in nutrient acquisition, were present in almost all the species depicted in **Figure 1**. The main differences are that strain B10502 lacks the *hbl* operon and *ilsA*, whereas strain T1 lacks *clpC* and *clpP* and the enhancin (*mpbE*) genes (**Figure 1**, **Supplementary Figure 2**). Notably, although B10502 is genetically close to *B. anthracis*, it lacks the genes involved in anthrax disease (*pag*, *lef*, and *cap*). Of note is that the genomic analyses confirmed the absence of any *cry* genes in both strains.

### *G. mellonella* infection

**Figure 2:**
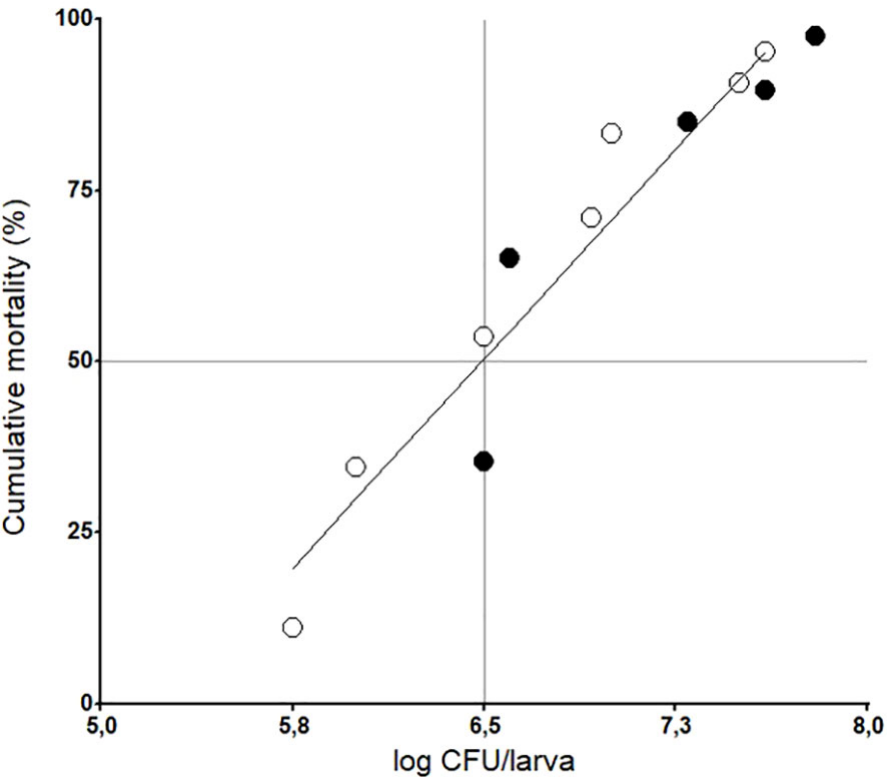
Dose-dependent cumulative mortality of *Galleria mellonella* following oral infection with *Bacillus cereus* strains T1 (*empty circle*) or B10502 (*filled circle*). Larvae were co-administered bacteria with the Cry1Ca toxin (3 µg/larva) by gavage. Data show mortality at 24 h post-infection (PI) from a representative experiment (*n* = 20 larvae per group).

Having identified distinct phylogenetic positions and virulence gene profiles, we next investigated whether these strains exhibit differential pathogenicity in the *G. mellonella* infection model. Both strains induced dose-dependent mortality in *G. mellonella* larvae following oral inoculation (**Figure 2**). To potentiate the biological effect of the strains, the Cry1Ca toxin was co-administeredwith the bacteria, as neither toxin nor bacteria administered alone caused significant mortality(Salamitou et al., 2000). Indeed, only a 10% mortality rate was observed with Cry1Ca (3 µg/larva) alone (**Supplementary Figure 3**).

**Figure 3:**
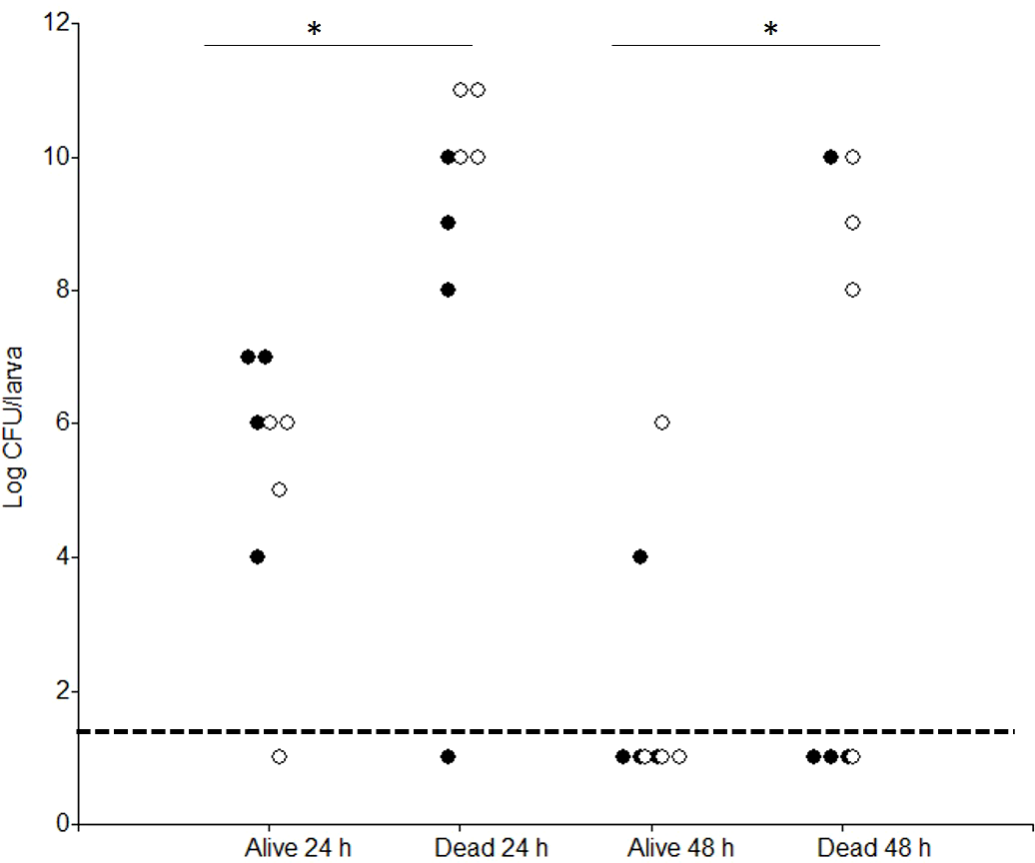
Persistence of total bacteria (spores plus vegetative forms) in alive or dead *Galleria mellonella* larvae at different time points (24 and 48 h) after oral infection with *Bacillus cereus* T1 (empty circle) or B10502 (filled circle). In all cases, larvae were inoculated with vegetative cells at doses corresponding to an ED_50_ (lethal dose 50%; log 6.5 CFU/larva). Data represent the results from four independent experiments (two larvae per experimental point). Values below the dotted line (1.6 log CFU/larva) were below the detection limit. *CFU*, colony-forming units.

To quantitatively evaluate the virulence of the strains, data on cumulative mortality (in percent) were analyzed at different infection doses. Firstly, we determined that a single, unified dose–response curve provides an adequate fit to the data as the more complex two-curve model did not offer a statistically significant improvement (*p* > 0.05). Based on this unified model, the estimated ED_50_ was log 6.5, a single value that applies to both the T1 and B10502 strains. To better understand the fate of the two *B. cereus* strains following oral infection, we then quantified the bacterial loads in infected larvae over time. The results indicated that, for both strains, bacterial counts were significantly (*p* < 0.05) higher in dead larvae than in surviving larvae (**Figure 3**). After 48 h, the bacterial loads were generally below the detection limit in surviving larvae (**Figure 3**).

To assess sporulation, thermoresistant bacteria (TR) were quantified. Spores were undetectable (below 50 CFU/larva, 1.6 log) in surviving larvae (data not shown). In dead larvae, no TR forms were detected for strain B10502, while spores were detected only for T1 at both 48 and 144 h PI in infected larvae (**Table 1**). No *B. cereus-*like colonies were observed in uninfected larvae.

**Table 1:** Thermoresistant bacteria (spores) in dead larvae at various time points post-infection by gavage at ED_50_ dose for each *Bacillus cereus* strain (log 6.5 CFU each).

Strain	Time post-infection (h)		
	24	48	144
T1	<1.6	8.6 ± 0.1	7.5 ± 0.9
B10502	<1.6	<1.6	<1.6

Values are expressed as log_10_ (CFU/larva ± SD).

### Melanization response in *G. mellonella* and phenoloxidase activity following *B. cereus* infection

**Figure 4:**
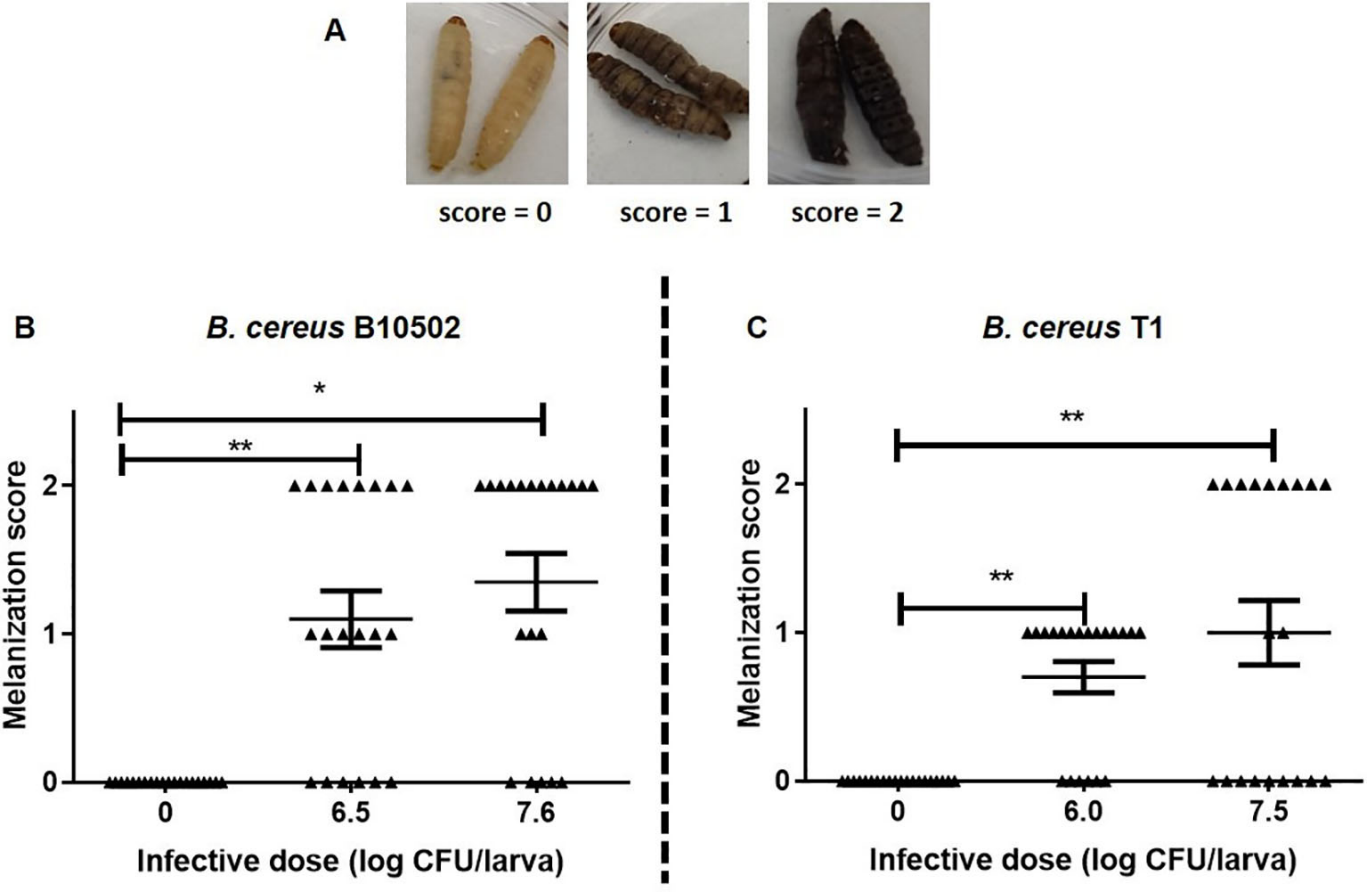
Melanization response of *Galleria mellonella* larvae following oral infection with *Bacillus cereus* strains T1 and B10502. **(A)** Representative images illustrating the melanization scoring system: score 0 (uninfected control) and two representative levels of melanization. **(B, C)** Melanization scores (filled triangle) recorded 24 h post-gavage with vegetative cells of strain T1 **(B)** or B10502 **(C)** in the presence of the Cry1Ca toxin. Asterisks indicate statistically significant differences relative to the uninoculated control [phosphate-buffered saline (PBS), zero bacteria]. *p < 0.05, **p < 0.01 (Bonferroni’s multiple comparisons test). Results are representative of three independent experiments (*n* = 20 larvae per group).

To investigate the immune defense mechanisms triggered by the infection, the melanization reaction in larvae was assessed as an indirect marker of the activation of the PO system, a key component of the insect innate immune response against infection. **Figure 4** shows the melanization scores of larvae orally infected with two different doses of *B. cereus* strain T1 or B10502. In all infected groups, dead larvae showed a dose-dependent score of melanization response (**Figures 4B, C**). Scores 1 and 2 correspond to dead larvae. Control larvae, which were inoculated with PBS + Cry1Ca, showed no melanization (score = 0).

**Figure 5:**
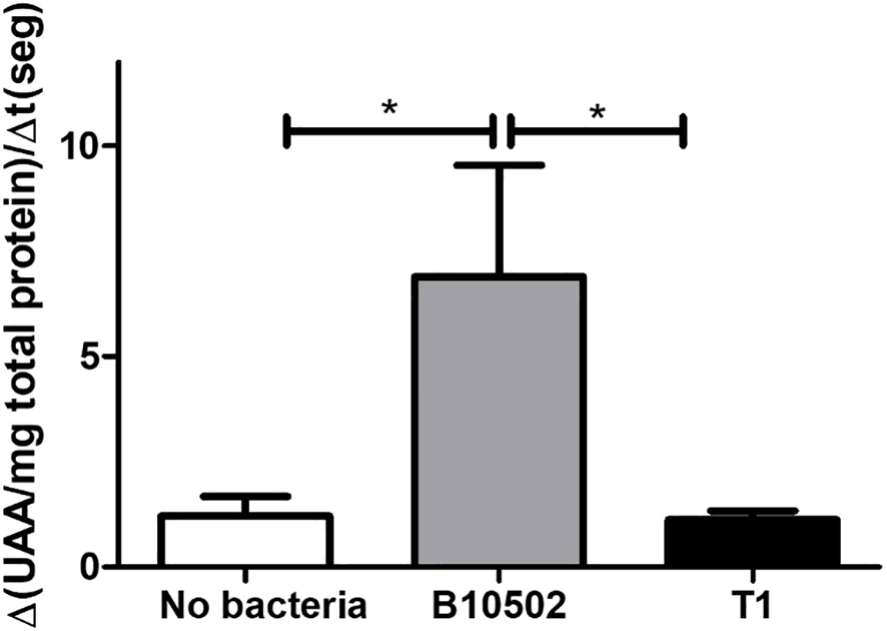
Phenoloxidase activity in the hemolymph of larvae at 4 h post-infection (PI) with an ED_50_ dose of *Bacillus cereus* B10502 or T1 vegetative cells via oral gavage along with the Cry1Ca toxin. Controls received only phosphate-buffered saline (PBS). Results are expressed as the rate of change in arbitrary units of absorbance per milligram total protein per second (arbitrary absorbance units/mg protein) × s^−1^). *Asterisks* indicate statistically significant differences relative to the uninoculated control (no bacteria). *p < 0.05. Values represent the variation of absorbance arbitrary units (AAU) per milligram of protein with respect to time. Results are averages (*n* = 3) from a representative experiment of two independent experiments.

To gain further insight into the melanization response, the PO activity in hemolymph was measured at 4 h PI to capture early enzymatic activation preceding visible melanization using an ED_50_ dose (**Figure 5**). The quantification of PO revealed that infection with B10502 significantly elevated the PO levels, whereas the values for larvae infected with strain T1 were similar to those of the uninfected control group.

**Figure 6:**
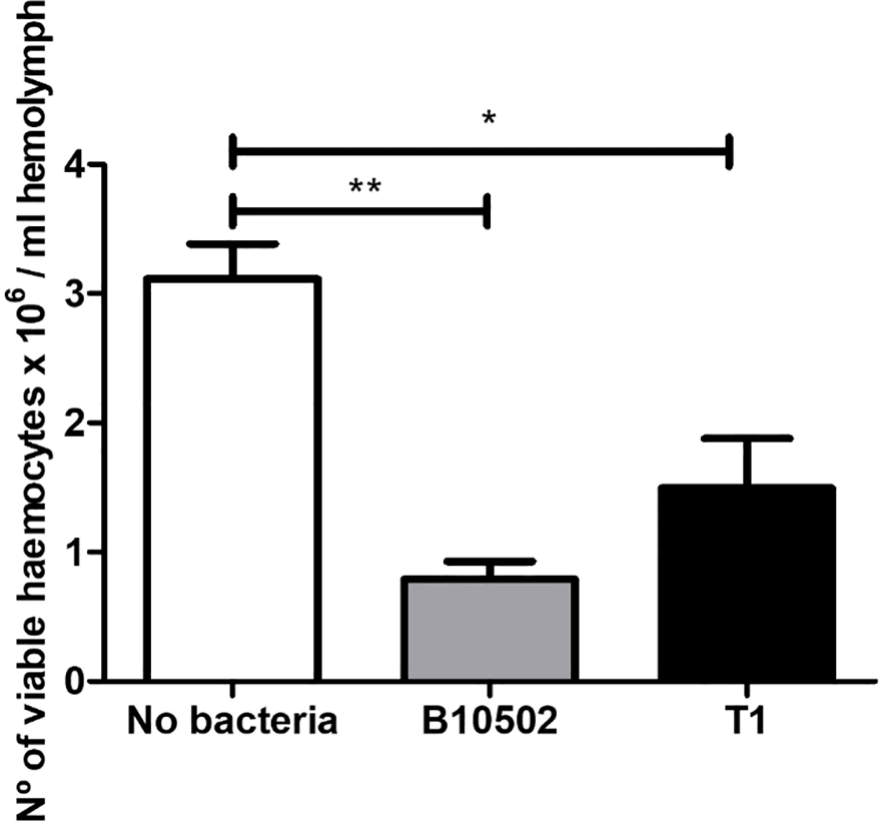
Viability of hemocytes 4 h after oral infection with vegetative *Bacillus cereus* T1 and B10502 strains. The infection dose was log 6.5 CFU/larva. Controls received phosphate-buffered saline (PBS) plus the Cry1Ca toxin. *Asterisks* indicate statistically significant differences relative to the uninoculated control (no bacteria). *p < 0.05, **p < 0.001 using Tukey’s test. Results show averages (*n* = 3) of a representative experiment from two independent experiments.

Since the above findings suggest an impact on the larval defense mechanisms following infection, we next investigated the cellular component of the immune response by assessing hemocyte survival after oral challenge. Both strains significantly reduced the number of viable hemocytes relative to the uninfected controls (**Figure 6**). These data indicate that both strains deplete circulating hemocytes, potentially through nodulation or cytotoxicity, with B10502 eliciting a significantly stronger effect.

## Discussion

Members of the *B. cereus* group display a wide spectrum of virulence mechanisms through the secretion of extracellular toxin factors to direct the interaction with eukaryotic cells (Minnaard et al., 2001, 2004, 2013; López et al., 2013; Castiaux et al., 2016; Gao et al., 2020). Using the *G. mellonella* model, it has been shown that infection with *B. cereus* is capable of killing larvae through either oral or hemocoel infection at 37°C (Salamitou et al., 2000; Stenfors Arnesen et al., 2011).

In this study, the *G. mellonella* model was used to dissect the strain-specific pathogenic mechanisms of two foodborne *B. cereus* strains, i.e., B10502 and T1, which display different biological activities on cultured human cells (Minnaard et al., 2007, 2013; Rolny et al., 2017). Specifically, strain T1 has been shown to invade cultured human enterocytes, while the virulence of strain B10502 appears primarily associated with the production of extracellular factors (Minnaard et al., 2004). This model, which bridges invertebrate innate immunity with mammalian-like defense responses, provided insights into how bacterial genomic differences may possibly translate into distinct infection phenotypes.

Genomic and phylogenomic analyses of strains T1 and B10502 (**Figure 1**; **Supplementary Figures 1**, **2**) positioned B10502 within genetic group III (*B. anthracis*-like) and T1 within group IV (*B. cereus/B. thuringiensis sensu stricto*), consistent with previously defined *B. cereus* clades (Guinebretière et al., 2008). Although both genetic groups III and IV include strains capable of infecting vertebrates, they have different patterns of host association and varying ability to cause food poisoning. Indeed, published data reported that only 29% of the 866 isolates in the clade to which group IV (*B. cereus s.s.* and *B. thuringiensis*) belongs were associated with vertebrate infections compared with 72% of the 548 isolates in the clade to which group III belongs (Raymond and Federici, 2017). Despite the proximity of B10502 to *B. anthracis*, it lacks the canonical anthrax toxin and capsule genes involved in anthrax disease (*pag*, *lef*, and *cap*) and does not carry the genes responsible for emetic toxin production, implying that its virulence relies on alternative mechanisms such as phospholipases, cytotoxins, and immune modulators. Both strains share a conserved core of virulence determinants (e.g., Nhe, CytK, and InhA metalloproteases), but each lacks specific loci that may explain the divergent infection outcomes. B10502, which is deficient in *hbl* and *ilsA* (related to membrane pore formation and iron acquisition, respectively), might compensate through stronger induction of host immune activation and oxidative stress responses, whereas T1, which is missing *clpC/clpP* (ATP-dependent protease system for stress adaptation and protein repair) (Sornchuer et al., 2023) and *mpbE* (enhancin, a metalloprotease implicated in peritrophic matrix degradation) (Hajaij-Ellouze et al., 2006), exhibits lower PO activation but greater persistence, suggesting enhanced immune evasion and sporulation capacity.

Taken together, these results support the hypothesis that the *B. cereus* group members share a common ancestor from which various virulence traits have been acquired, inherited, and differently maintained or lost (Sabin et al., 2024).

A majority of the virulence assays conducted in this study showed strain-dependent effects in the *G. mellonella* model, as previously observed in other systems. When co-administered orally with Cry1Ca, both *B. cereus* strains exhibited a similar dose–response effect and induced similar mortality, whereas neither bacteria nor Cry1Ca toxin alone induced mortality in *G. mellonella*, confirming that larval mortality in *G. mellonella* requires epithelial disruption (Salamitou et al., 2000). However, T1 persisted in the host and produced spores, whereas B10502 did not, suggesting differences in adaptation to the insect environment. It is known that the presence of the trypsin-activated Cry1Ca toxin during oral infection with entomopathogenic *Bacillus* species leads to bacterial accumulation near the epithelium, facilitating bacterial translocation into the hemocoel, where subsequent proliferation results in lethality (Consentino et al., 2021). Interestingly, oral administration of high doses of strain T1 (approximately 10^7^ bacteria per larva) caused 40% mortality even without the Cry1Ca toxin (data not shown), suggesting that this strain possesses intrinsic virulence or host adaptation factors that make it more infectious.

Injection into the hemocoel revealed that T1 could kill larvae at lower concentrations thanB10502 (**Supplementary Figure 4**), consistent with previous *in vitro* observations of the invasive properties of T1 (Minnaard et al., 2004).

Strain T1 reached high levels in dead larvae, with all bacteria found as spores within 144 h PI, whereas strain B10502 was undetectable at this time point. This capacity of the T1 strain to produce spores *in vivo* postmortem for long-term persistence likely confers it with an ecological advantage and may be related to differences in the stress response pathways, including oxidative stress regulators, and the sporulation control genes. Conversely, the inability of B10502 to form spores *in vivo*, despite sporulating *in vitro*, may reflect hypersensitivity to the insect oxidative defenses, including PO activity. In addition, the observed differences between these strains may reflect alterations in host gene regulation during the infection process.

The host immune response is crucial in the control of microbial spread after infection. Due to their lack of adaptive immunity, insects respond to microbial stimuli through both humoral and cellular defense mechanisms (Grizanova et al., 2014). Humoral responses include clotting, melanization, and the release of antimicrobial peptides, while cellular responses involve phagocytosis, nodulation, and encapsulation (Kavanagh and Reeves, 2004). In insects, the PO pathway can be considered functionally equivalent to the mammalian complement system (Gillespie et al., 1997). Melanin synthesis and deposition followed by the encapsulation of pathogens, coagulation, and opsonization are key steps in the immunological response to infection (Pereira et al., 2018). Furthermore, the activation of the PO cascade leads to the production of hydrogen peroxide and superoxide, two key components of the reactive oxygen (ROS) and nitrogen species (NOS) (Komarov et al., 2005). Oral infection with both *B. cereus* strains reduced circulating hemocytes (**Figure 6**), consistent with nodulation-mediated cell recruitment and phagocytosis (Pereira et al., 2018). B10502 induced higher PO activity (**Figure 5**), correlating with melanization and stronger humoral responses, whereas T1 evaded humoral defenses, consistent with its persistence and sporulation. Indeed, when PO activity was assessed in infected larvae, strain-specific differences were observed.

Infection with B10502 elicited strong PO activation and a pronounced hemocyte depletion, whereas strain T1 elicited weaker melanization and achieved longer survival within the host (**Figure 5**). These strain-specific differences in colonization dynamics, sporulation capacity, and immune modulation may be correlated with the distinct virulence gene profiles. Notably, the host response to strain B10502 is consistent with previous studies showing that intestinal infection with *B. thuringiensis* enhances PO activity and lysozyme-like activity in the hemolymph, while it downregulates certain immune responses, resulting in decreased phagocytic activity of hemocytes (Grizanova et al., 2014). These findings indicate that, although strain B10502 is genetically more closely related to *B. anthracis*, it behaves more like *B. thuringiensis* in terms of immune activation. In contrast, strain T1, which is more closely related to *B. cereus sensu stricto* and *B. thuringiensis*, does not elicit the same humoral response. These genomic differences between strains provide a rationale for their distinct infection outcomes. B10502 lacks *hbl* and *ilsA*, which may reduce virulence and impair adaptation to low-iron conditions *in vivo*, but it induces strong melanization and PO activation, likely contributing to hemocyte depletion. T1, which lacks *clp* and *mpbE*, appears to downregulate humoral immunity, facilitating survival, higher bacterial counts, and spore formation. Interestingly, the Clp protease plays a critical role in bacterial stress response, and disruption of the ClpP function has been shown to impair virulence and survival in several pathogenic bacteria (Yamamoto et al., 2001; Frees et al., 2003).

The divergent strategies identified (persistence in T1 *vs*. aggression in B10502) likely reflect a complex coordination between the core genome and the mobilome (plasmids, prophages, and insertion sequences). Although the specific contribution of extrachromosomal elements to these phenotypes remains to be fully elucidated, our findings suggest that the presence of toxin genes alone is insufficient to predict clinical outcomes. This underscores the importance of the host–pathogen interface in defining the pathogenic success of a strain, regardless of the genetic vehicle (plasmid or chromosome) of its virulence determinants.

Further studies are required to determine the origin of their distinct pathogenic profiles. Nonetheless, our findings, combining genomic and infection data, highlight the utility of the *G. mellonella* infection model for the evaluation of the pathogenic potential of *B. cereu*s strains and for the study of the mechanisms involved in *B. cereus* pathogenicity and the interplay between host immunity and bacterial adaptation; they suggest that subtle gene content differences can markedly alter the infection trajectory in *G. mellonella*. This model also provides valuable insights into the conserved immune mechanisms across vertebrates and invertebrates and offers a useful platform for the dissection of *B. cereus* virulence diversity following oral route administration.

The results of the present research show that the infection of *G. mellonella* through the oral route offers further insight into the effect of *B. cereus* strains isolated from foods. While high bacterial doses lead to devastating, uniform effects on the larvae, the inoculation of lower bacterial concentrations allows the detection of subtle host immune responses that can effectively discriminate between microorganisms at the strain level. By employing *G. mellonella*, we demonstrate for the first time that two strains isolated from food poisoning outbreaks can trigger diametrically opposed host responses: a “persistence–evasion” strategy (T1 strain) *versus* an “aggressive–inflammatory” strategy (B10502 strain). This shifts the focus of *B. cereus* research from the cataloging of simple virulence factors to the study of complex colonization dynamics.

Our results challenge the notion that the presence of toxin genes is the sole predictor of pathogenicity; instead, they underline how colonization dynamics and host response drive clinical outcomes. The *G. mellonella* model establishes itself not only as a screening tool but also as a valuable biosensor for identifying phenotypic variants that *in vitro* systems fail to detect. This provides a foundation for future research on the co-evolution of opportunistic pathogens and the host immune response. In this context, the differences in the *in vivo* sporulation between the studied strains represent a critical biological finding, as sporulation is a key mechanism for pathogen dissemination. In addition, while both strains reduced hemocyte concentration, only strain B10502 activated the PO cascade, suggesting previously unknown strain-dependent immune evasion mechanisms.

These findings encourage future research using this model to elucidate both the virulence profiles of foodborne *B. cereus* and the specific defense mechanisms of the host against the pathogen.

## Data Availability

The data presented in this study are deposited at https://www.ncbi.nlm.nih.gov/sra/PRJNA1115104. Raw Illumina sequence data for B. cereus strains B10502 and T1 are available in the NCBI-SRA (Short Read Archive) repository under accession numbers SRR34420510 and SRR34420511, respectively. The Whole Genome Shotgun project has been deposited at DDBJ/ENA/GenBank with the accession numbers JBPPSQ000000000 for B. cereus B10502 and JBPPSP000000000 for *B. cereus* T1. Details on genomic analysis are provided in **Supplementary Table 1** in the **Supplementary Material**.

## Supplementary material

Supplementary Figure 1 Phylogenetic ANI positions of *Bacillus cereus* B10502 and T1 strains indicated by grey arrows within the GTDB representative group of *Bacillus cereus sensu lato* species.

Supplementary Figure 2 Phylogenetic positions of B10502 and T1 strains indicated by grey arrows within the group of GTDB representative genomes of *B. cereus sensu lato* species as well as the presence/absence status of a subset of virulence or adaptation genes relevant to pathogenesis in mammalian and insect models (detailed information of corresponding Vir-gene panggolin clusters is presented in Supplementary Table 1. Gene subset profiles of *B. cereus* B10502, *B. cereus* T1, and their closest relatives (based on ANI) are indicated by dashed rectangles.

Supplementary Figure 3 Kaplan-Meier survival curves of *G. mellonella* infection by gavage with *B. cereus* strains. Activated Cry1Ca toxin from *B. thuringiensis* was coadministered with bacteria at 3 µg/larva. Doses used (log CFU/larva) for T1 strain **(A)**: 5.7 (□), 6.1 (∆) and 7.5 (◊), for B10502 strain **(B)**: 5.5 (□), 6.5 (∆) and 7.6 (◊). No-infected larvae (○) were administered with PBS containing 3 µg activated toxinCry1Ca /larva. Different letter represents significant differences (Gehan-Breslow-Wilcoxon test; p<0.0001). The data show a representative experiment.

Supplementary Figure 4 Lethality of *Galleria mellonella* infection by injection in the haemocoel with vegetative cells of B. cereus strains T1 (black circles) or B10502 (grey squares) at different doses. The data show mortality percentages at 24 h post-infection of a representative experiment.
