# Supplementary figures and images for: Pathogenesis of human-derived Bacillus cereus strains: lessons from the insect Galleria mellonella immune responses
Source: Front Cell Infect Microbiol. 2026 Apr 15;16:1698447. doi: 10.3389/fcimb.2026.1698447 (PMC13124694; doi:10.3389/fcimb.2026.1698447)

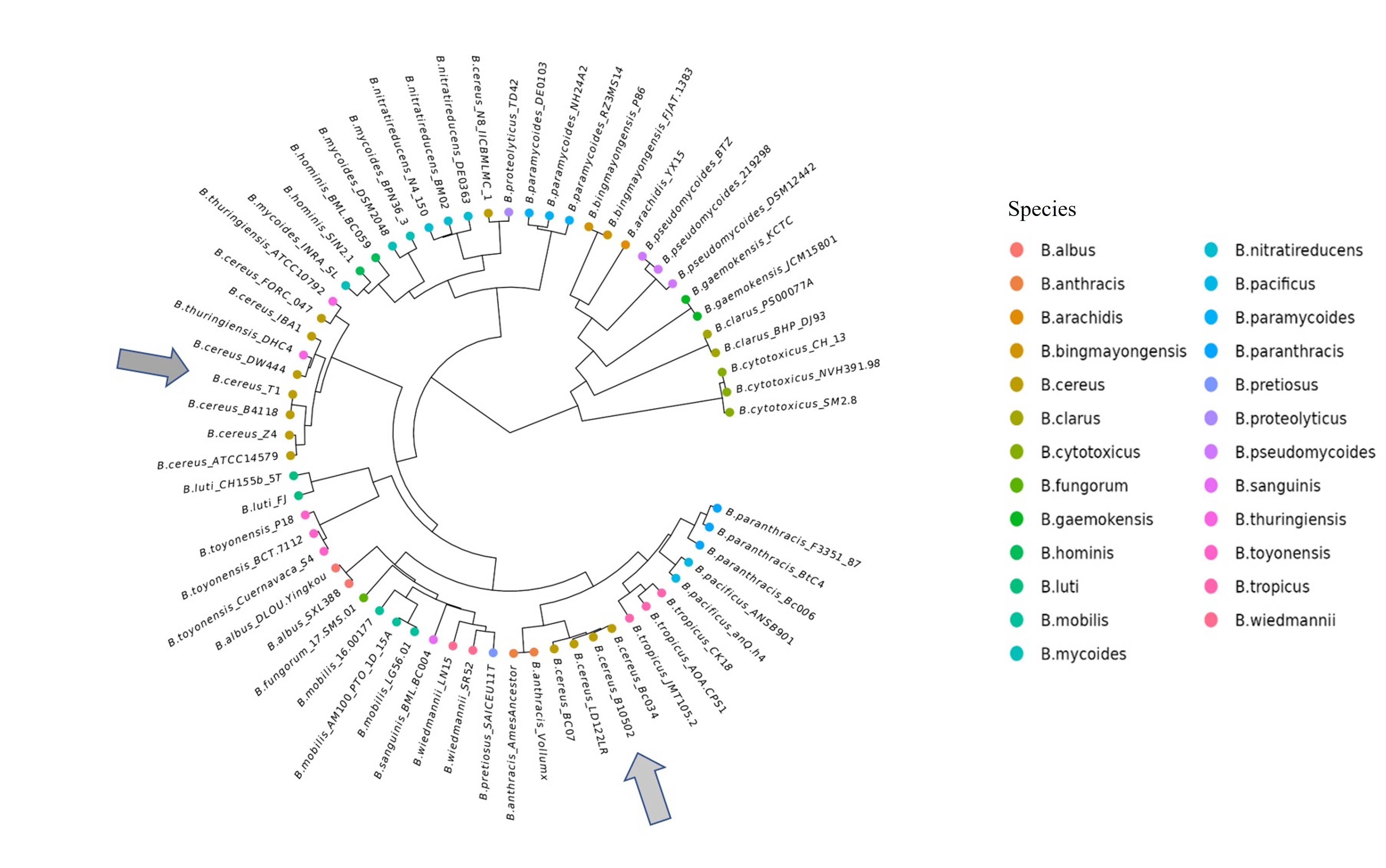

Supplement: Supplementary Figure 1 — Phylogenetic ANI positions of Bacillus cereus B10502 and T1 strains indicated by grey arrows within the GTDB representative group of Bacillus cereus sensu lato species. [file Image1.jpeg]

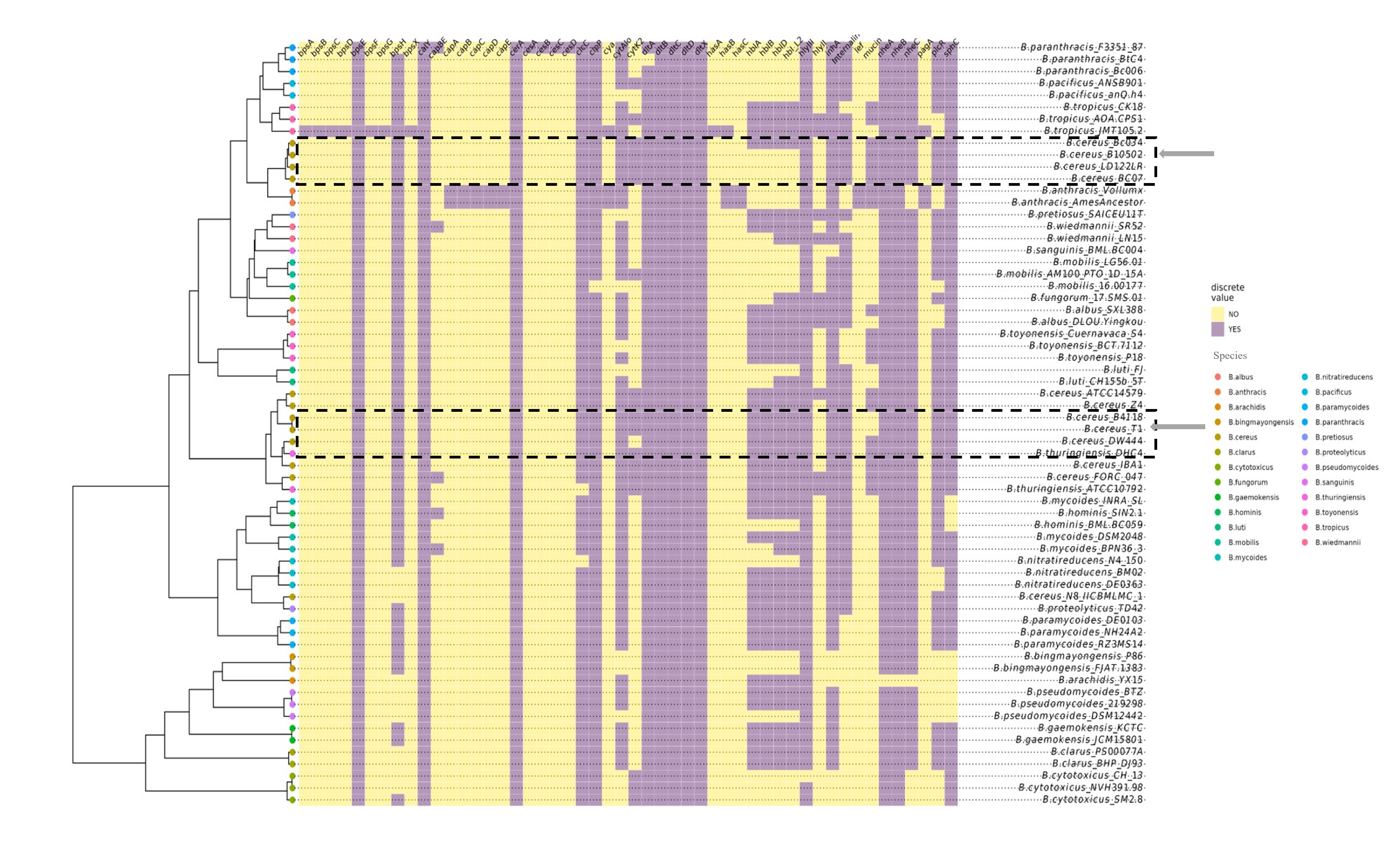

Supplement: Supplementary Figure 2 — Phylogenetic positions of B10502 and T1 strains indicated by grey arrows within the group of GTDB representative genomes of B. cereus sensu lato species as well as the presence/absence status of a subset of virulence or adaptation genes relevant to pathogenesis in mammalian and insect models (detailed information of corresponding Vir-gene panggolin clusters is presented in Supplementary Table 1. Gene subset profiles of B. cereus B10502, B. cereus T1, and their closest relatives (based on ANI) are indicated by dashed rectangles. [file Image2.png]

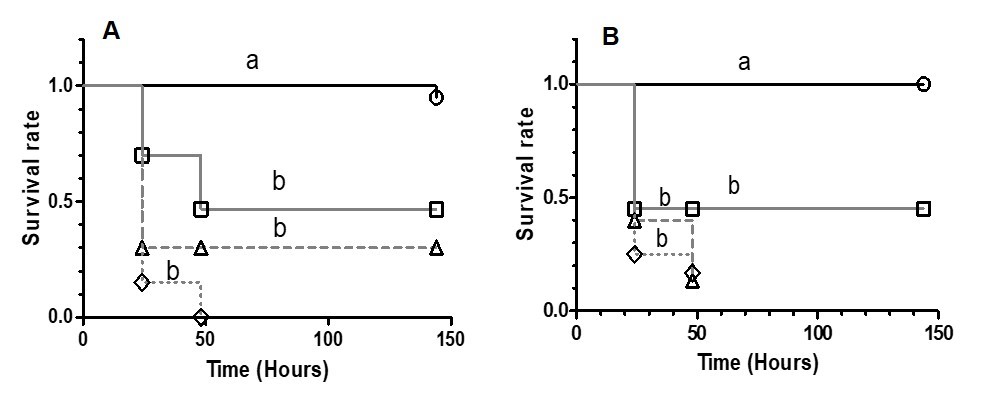

Supplement: Supplementary Figure 3 — Kaplan-Meier survival curves of G. mellonella infection by gavage with B. cereus strains. Activated Cry1Ca toxin from B. thuringiensis was coadministered with bacteria at 3 µg/larva. Doses used (log CFU/larva) for T1 strain (A): 5.7 (□), 6.1 (∆) and 7.5 (◊), for B10502 strain (B): 5.5 (□), 6.5 (∆) and 7.6 (◊). No-infected larvae (○) were administered with PBS containing 3 µg activated toxinCry1Ca /larva. Different letter represents significant differences (Gehan-Breslow-Wilcoxon test; p<0.0001). The data show a representative experiment. [file Image3.jpeg]

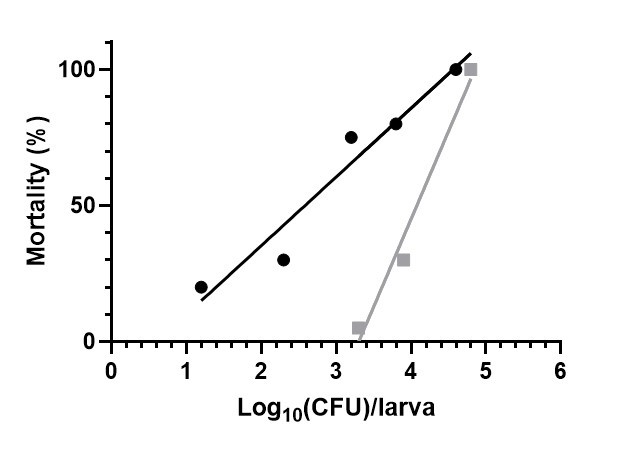

Supplement: Supplementary Figure 4 — Lethality of Galleria mellonella infection by injection in the haemocoel with vegetative cells of B. cereus strains T1 (black circles) or B10502 (grey squares) at different doses. The data show mortality percentages at 24 h post-infection of a representative experiment. [file Image4.jpeg]
