# Supplementary material for: Pathogenesis of human-derived Bacillus cereus strains: lessons from the insect Galleria mellonella immune responses
Source: Front Cell Infect Microbiol. 2026 Apr 15;16:1698447. doi: 10.3389/fcimb.2026.1698447 (PMC13124694; doi:10.3389/fcimb.2026.1698447)
Supplement: Supplementary file 7 [file Table2.docx]

 ST2: Functional categories and primary function of genes
