# Supplementary material for: Pathogenesis of human-derived Bacillus cereus strains: lessons from the insect Galleria mellonella immune responses
Source: Front Cell Infect Microbiol. 2026 Apr 15;16:1698447. doi: 10.3389/fcimb.2026.1698447 (PMC13124694; doi:10.3389/fcimb.2026.1698447)
Supplement: Supplementary file 8 [file Table3.docx]

**LEGENDS OF THE SUPPLEMENTARY FIGURES (SF)**

SF1: Phylogenetic ANI positions of *Bacillus cereus* B10502 and T1 strains indicated by grey arrows within the GTDB representative group of *Bacillus cereus sensu lato* species.

SF2: Phylogenetic positions of B10502 and T1 strains indicated by grey arrows within the group of GTDB representative genomes of *B. cereus* *sensu lato* species as well as the presence/absence status of a subset of virulence or adaptation genes relevant to pathogenesis in mammalian and insect models (detailed information of corresponding Vir-gene panggolin clusters is presented in Supplementary Table 1. Gene subset profiles of *B. cereus* B10502, *B. cereus* T1, and their closest relatives (based on ANI) are indicated by dashed rectangles.

SF3: Kaplan-Meier survival curves of *G. mellonella* infection by gavage with *B. cereus* strains. Activated Cry1Ca toxin from *B. thuringiensis* was coadministered with bacteria at 3 µg/larva. Doses used (log CFU/larva) for T1 strain (A): 5.7 (□), 6.1 (∆) and 7.5 (◊), for B10502 strain (B): 5.5 (□), 6.5 (∆) and 7.6 (◊). No-infected larvae (○) were administered with PBS containing 3 µg activated toxinCry1Ca /larva. Different letter represents significant differences (Gehan-Breslow-Wilcoxon test; p<0.0001). The data show a representative experiment.

SF4: Lethality of *Galleria mellonella* infection by injection in the haemocoel with vegetative cells of *B. cereus* strains T1 (black circles) or B10502 (grey squares) at different doses. The data show mortality percentages at 24 h post-infection of a representative experiment.
